# A hybrid stellar mass black-hole optimization framework for finding significant biclusters using average Kendall rank correlation

**DOI:** 10.1038/s41598-025-20501-z

**Published:** 2025-10-28

**Authors:** R. Balamurugan

**Affiliations:** School of Computer Science and Engineering (SCOPE), Vellore Institute of Technology, Vellore, 632014 Tamil Nadu India

**Keywords:** Clustering, Genes, Kendall correlation, Nelder-Mead, Coherent, Optimization, Levy flight, Bioinformatics, Gene expression analysis

## Abstract

Microarray gene expression data are high-dimensional and complex, with patterns that may appear only under specific conditions. Traditional clustering often misses these local patterns, whereas biclustering can reveal groups of genes with coordinated expression across particular conditions. In this paper, we propose a biclustering approach using average Kendall correlation, which captures nonlinear and monotonic relationships often overlooked by standard measures like Euclidean distance or Pearson correlation. To efficiently search for optimal biclusters, we implement a modified stellar mass black-hole optimization (MSBO) approach that integrates the Nelder–Mead simplex method with Lévy flight to enhance both local and global search capabilities. The proposed technique is validated on couple of widely used benchmark gene expression datasets, namely the yeast cell cycle and lymphoma. The biological importance of the identified biclusters is evaluated with the help of the gene ontology (GO) database. Experimental results demonstrate that our method outperforms traditional approaches in identifying statistically significant and biologically relevant biclusters, achieving a p-value of 3.73 × 10^−16^. These findings address the pressing need for more effective biclustering techniques in the study of microarray data.

## Introduction

**Fig. 1:**
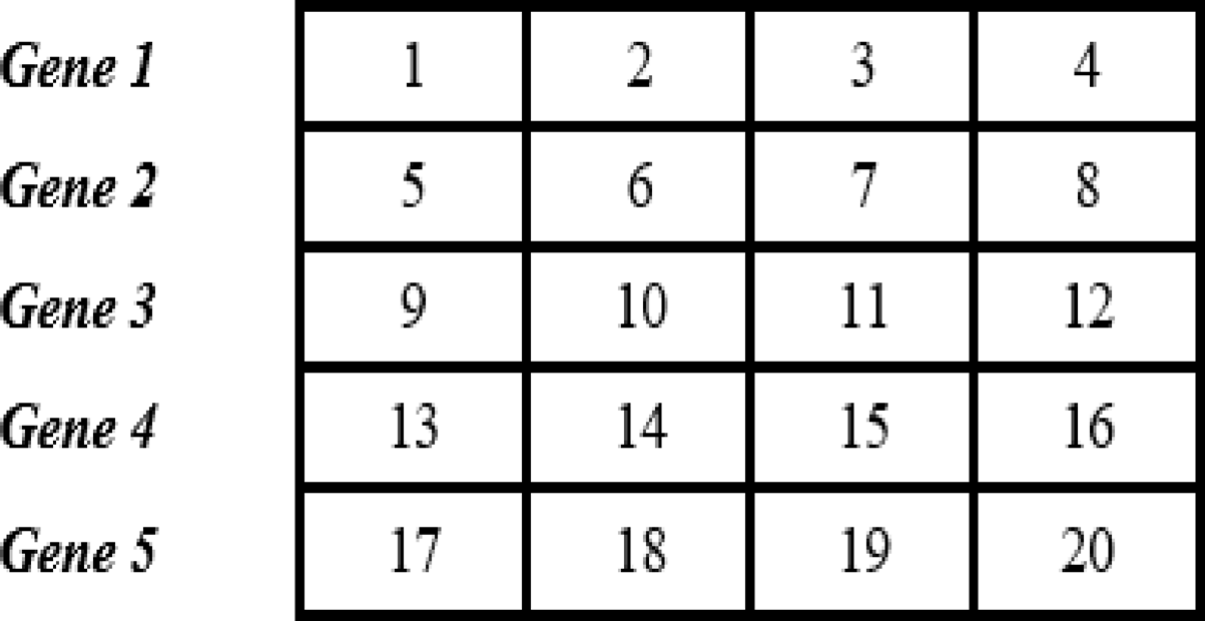
Example of a gene expression matrix.

Functional genomics studies how genes and proteins work and interact, and microarray technology helps by allowing scientists to examine thousands of genes at once to understand complex biological processes^1^. A DNA microarray consists of small DNA probes covalently attached to a chemical matrix and arranged on a solid surface, often representing individual genes^2^. Results from microarray studies are presented as gene expression datasets, typically comprising thousands of spots. These data are represented in the arrangement of a matrix, where rows represent individual genes, columns indicate experimental conditions, and each cell records the expression value of a gene for a specific condition^3^. The entries of this matrix are real numbers. An example of a gene expression matrix is depict in Fig. 1. Here, the number “1” shows the expression level of Gene 1 in the first condition, while the number “20” shows the expression level of Gene 5 in the fourth condition. By using this matrix, scientists can see how genes change across different conditions, notice which ones are more active or less active, and connect these patterns to important biological processes or diseases.

Data analysis methods help uncover patterns or classify data automatically, without the need for direct human input. The real challenge, however, is in developing methods that can effectively deal with the enormous volume of biological data being produced today^4^. One approach is unsupervised pattern classification, which groups similar items together while separating those that are different. This can be applied to observations, datasets, or feature vectors. Among these approaches, clustering has become one of the most widely used techniques in biology. It allows researchers to detect meaningful biological patterns, such as new disease subtypes, possible treatment strategies for cancers, and even genetic interaction networks^5^.

In the context of gene expression studies clustering is used to group genes or experimental conditions that display comparable expression trends across samples or time points. This approach is commonly classified into three types: gene-based (row) clustering, condition-based (column) clustering, and biclustering. Genebased clustering identifies groups of genes that exhibit similar regulation under different conditions. In condition-based clustering, groups of related conditions are identified by analyzing their patterns across all genes^6^. However, some genes may be significant only in specific conditions. Condition-specific marker genes need to be identified. They are important because they can indicate particular tissues or be linked to certain diseases. To capture this complexity, clustering must be performed in both dimensions at the same time. Biclustering addresses this by simultaneously grouping genes and conditions, providing deeper biological insights that traditional one-dimensional clustering often misses^7^.

Biclustering is a useful method for finding links between groups of genes and experimental conditions. It also helps reveal functional modules in biological networks, sort patients by their phenotypes, and study how genes relate to drugs. Beyond bioinformatics, biclustering is applied in many other areas, including text analysis, recommendation systems, and climate research. Cheng and Church^8^ coined the term “biclustering” for use with gene expression data. Clustering-based biclustering methods fall into two main groups. One group follows an iterative row-and-column strategy: it clusters the rows of the data matrix, clusters the columns by working on the matrix transpose and then combines the row and column clusters to form biclusters. The other group uses one-dimensional clustering techniques. Examples of the iterative row-and-column approach include Coupled Two-Way Clustering, Interrelated Two-Way Clustering, and Double Conjugated Clustering.

In contrast, One-Dimensional Clustering algorithms perform clustering on single dimension of the data matrix and then use heuristic strategies to combine the results into biclusters, as demonstrated in several studies. However, these methods are often sensitive to noise, fail to capture nonlinear patterns, and may produce suboptimal or redundant biclusters because they rely on greedy heuristics and the Mean Squared Residue (MSR) measure, which captures only linear coherence. Over time, numerous heuristic techniques have been proposed to identify biclusters, including ISA^9^, SAMBA^10^, HARP^11^, BicPAM^12^, Bimax^13^, and XMotifs^14^.

When working with gene expression data, biclustering usually aims to achieve two things: to find biclusters that are as large as possible and to keep the genes within them highly coherent. These two aims, however, are often in conflict^15,16^. As the size of a bicluster grows, the level of coherence generally drops. Finding biclusters is also computationally challenging. The problem is NP-complete because it involves searching through many possible combinations of gene subsets and condition subsets. With the rapid growth of data in genomics, transcriptomics, and proteomics, this task has become even more demanding.One way to manage this complexity is through metaheuristic approaches inspired by natural processes. Methods based on evolution, immune systems, ant colonies, or swarm behavior provide practical strategies for exploring large search spaces and have been widely applied to biclustering problems^17–24^.

Among nature-inspired methods, evolutionary computing has been the most widely adopted and has formed the basis for many biclustering algorithms. It offers flexible search capabilities for analyzing complex gene expression patterns but face major challenges such as high computational cost, sensitivity to parameter settings, and limited scalability. They often risk premature convergence and struggle to accurately detect overlapping or irregular biclusters common in biological data. Stellar mass black-hole optimization (SBO), a recent approach inspired by nature, has been put forth by Balamurugan et al.^25^. However, SBO for biclustering has several drawbacks, just like other meta-heuristic methods. To investigate patterns in gene expression data, we therefore present a modified SBO biclustering framework of the Nelder-Mead approach methods and levy flying with the benefits of both. This paper makes the following key contributions:

1. To enhance the population-based SBO algorithm’s adaptability across different search stages, the optimal black hole is chosen using the simplex approach from the original random population and adjustments to the structure of new individuals are made utilizing levy distribution operators.
2. In order to identify the most challenging biclusters that jointly feature scaling and shifting patterns, proposed method that utilizes average Kendall correlation measure as an objective function.

Next, we dive into the core of this paper. We’ll start by explaining a novel evaluation method specifically created to detect shifting and scaling patterns in biclusters. In section three, a brand-new hybrid SBO algorithm combining the Nelder-Mead approach with levy flight is described. The experiment using the new algorithm and the dataset for lymphoma and yeast is then described in section four. Section five is where the conclusion is made.

### Stellar-mass black hole optimization (SBO)

The SBO technique is a heuristic from nature that draws on the physical behaviour of stellar-mass black holes in universe. In this framework, each candidate solution is modeled as a black hole, with its fitness value corresponding to its “mass”. Better solutions are represented as more massive black holes. The algorithm operates through interactions among these black holes. Stronger (more massive) solutions absorb weaker ones, allowing the search to exploit the best candidates identified so far. The SBO prevents premature convergence by maintaining diversity within the search space. It applies a mechanism inspired by Hawking radiation, where weaker black holes are eliminated and substituted with new candidate solutions from unexplored regions. This cycle of removal and replacement forms a balanced and adaptive search strategy. This balance between exploration and exploitation makes SBO effective for solving complex, nonlinear, and high-dimensional optimization problems.

In this method, every candidate solution is treated as a black hole, and its quality is measured through a objective function *f*_*i.*_ The growth behavior of each black hole is governed by its ability to absorb matter, where higher fitness corresponds to greater mass and stronger attraction. The probability of a black hole *i* attracting others is given by its normalized fitness ratio $\:{\rho\:}_{i}=\frac{{f}_{i}}{\sum\:_{j=1}^{N}{f}_{i}}$, and its radiation emission is modelled by $\varepsilon _{i} = 1 - \rho _{i}$, representing the energy loss due to hawking radiation. The position of a black hole in the solution space is updated by considering both its growth (*Δa*_*i, d*_) and radiation (*Δe*_*i, d*_ ) across dimensions *d* using the update rule:

1 $$b_{{i,d}} \left( {t + 1} \right) = b_{{i,d}} \left( t \right) + \Delta a_{{i,d}} (t) - \Delta e_{{i,d}} (t)$$

*b*_*i*,d_ - Value of *i*^th^ Black hole in *d*^th^ dimension.

*Δa*_*i, d*_ - Growth at dimension *d* in *i*^th^ Black hole.

*Δe*_*i, d*_ - Radiation at dimension *d* in *i*^th^ Black hole.

T - Time epoch.

Merging of black holes is also simulated to maintain diversity and avoid premature convergence using:

2 $$b_{{k,d}} \, = \,rand() \times (b_{{p,d}} + \,b_{{q,d}} )$$

Where *b*_*p*_ and *b*_*q*_ ​ are neighboring black holes. The algorithm iteratively updates the population, replacing low-fitness black holes and encouraging exploration through stochastic emission and collision events.

SBO performs well compared to algorithms like Particle Swarm Optimization (PSO), Cuckoo Search Optimization (CSO), and the Firefly Algorithm (FA). It has been used in bioinformatics for biclustering microarray data. Naturally, it has ability to find biologically meaningful biclusters with good coverage and coherence. SBO’s adaptive design and self-balancing nature help it remain robust, scalable, and capable of escaping local optima. Since it is a relatively new algorithm, SBO has strong potential for future improvements and for use in hybrid methods for multi-objective and dynamic optimization.

### Nelder-Mead’s simplex method

The Nelder–Mead (NM) algorithm, created by John Nelder and Roger Mead^26^, is a popular approach for solving optimization problems. Because it is easy to apply, it is commonly used in scientific and engineering fields, including chemistry and medicine. One of its main advantages is that it does not require derivatives, which makes it suitable for problems with noisy or irregular data, as often seen in experiments and statistical applications. The method works by testing points within a geometric shape called a simplex (a triangle in two dimensions). At each step, it identifies the worst point and replaces it with a better one by reflecting it across the opposite side. If the new point shows significant improvement, the simplex is expanded in that direction. If the improvement is minimal, the simplex is shrunk toward the best solution identified up to that point. This process continues, with the simplex shrinking step by step, until the method converges toward the optimal solution.

### Workflow of Nelder-Mead simplex method


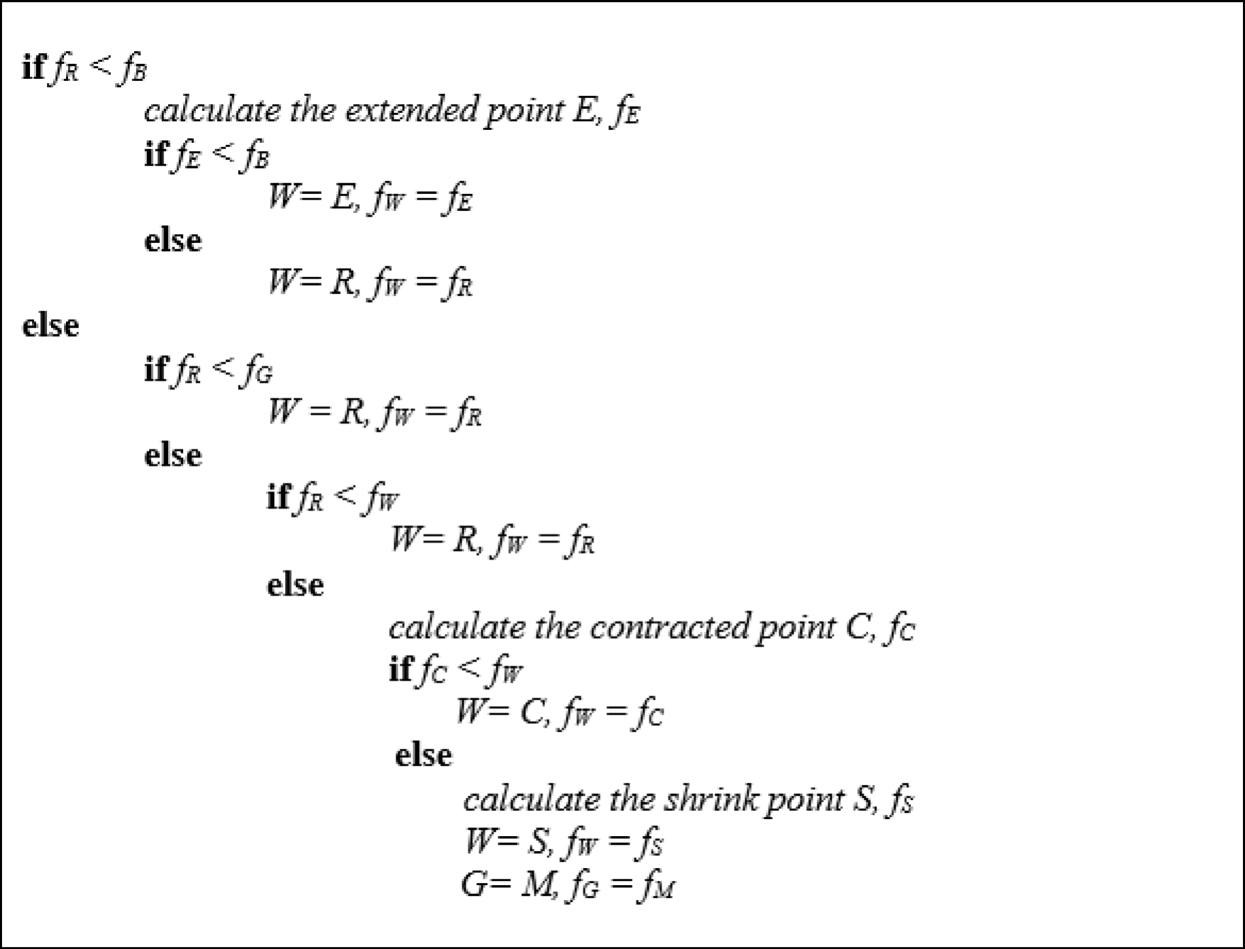


In the above snippet *f*_*B*_, *f*_*G*,_ *f*_*M*_, *f*_*W*_, *f*_*R*_, *f*_*E*_ *f*_*C*_ and *f*_*S*_ represent the fitness value of the points best *B*, good *G*, centroid *M*, worst *W*, reflection *R*, expansion *E*, contraction *C* and shrink *S* respectively.

Each step of the NM technique is composed of three key phases:

1. Ordering: First, rank the vertices of the current simplex based on their function values. Identify the best (B), second-best or good (G), and worst (W) points such that f(B) ≤ f(G) ≤ f(W).
2. Centroid calculation: Compute the centroid (M) of the best and good points, excluding the worst one.
3. Transformation: A superior point obtained by reflection, expansion, or contraction with respect to the optimal face is then used to update the working simplex by replacing its least favorable vertex. No more than two candidate points are evaluated in a single iteration, and they are limited to the line joining B and M. Upon acceptance, a candidate takes the place of the simplex’s poorest vertex. The simplex is contracted toward the best vertex, B, if none of the trial points improve, and n new vertices are calculated as a result.

### Correlation-based evaluation of biclusters

When rows and columns are grouped together into smaller submatrices within a larger dataset, an unsupervised data analysis technique called biclustering is used to find significant patterns. These sub matrices are interpreted using both descriptive and numerical measurements. To check how consistent a bicluster is, most biclustering methods rely on an evaluation function. One major challenge in evaluating biclusters from microarray gene expression data is the absence of ground truth and standard benchmarks, which makes it difficult to fairly measure their quality and biological significance. Among the commonly used measures, the Mean Squared Residue (MSR) function is popular^8^, but it has limitations in accurately reflecting the quality of a bicluster. Similarly, Wassim Ayadi^13^ introduced the Average Spearman Rank (ASR) function for this purpose, but its performance is also prone to errors. The primary goal of this research is to identify complex biclusters that exhibit both scaling and shifting patterns. Such a pattern-based relationship between pairs of genes can be expressed mathematically using Eq. 3.

3 $${\text{r}}_{{\text{Y}}} = ~\alpha + ~\beta \;\;\;\alpha ,~\beta ~ \in ~\mathbb{R}$$

Where r_X_ and r_Y_ denote the expression values of genes X and Y respectively. The parameter α represents the scaling factor, capturing proportional or monotonic changes between the two genes, while β accounts for a constant offset.

Using correlation-based measures to find biclusters with these patterns can be a valuable fitness function, as two genes with scaling and shifting patterns are usually linearly reliant on each other. One of the non-parametric correlation coefficients is Kendall’s tau^27^. The outcome of this measure is exactly the same as Spearman’s Rho and the Pearson correlation. Since it ranges from − 1 to + 1, it has the same characteristics as these other methods: its direction is defined by the sign (- +), and it has a range. The strength of the relationship increases with the value’s proximity to -1 or + 1. It assesses how similar two genes of different rankings are to one another. To determine the degree of linear dependency between two genes, M and N, the correlation coefficient is used. Let M1, … to define it. Assume that Mn is a sample for gene M and N1 is a sample of the same size n. Choosing different pairs (Mi, Ni) and (Mj, Nj) can be done in C(n,2). Use the following definitions to determine whether a pair is concordant, discordant, or neither for any such assignment.

Correspondent if (Mi > Mj and Ni > Nj) or (Mi < Mj and Ni < Nj).

Frictional if (Mi > Mj and Ni < Nj) or (Mi < Mj and Ni > Nj).

Pairs where Mi = Mj or Ni = Nj (i.e., ties) are excluded .

Let C represent the count of concordant pairs and D the count of discordant pairs; Kendall’s tau can then be expressed as:

4 $$\:\tau\:=\frac{C-D}{C(n,2)}$$.

Consider a bicluster (M′, N′) within a data matrix A(M, N). The Average Kendall Rank (AKR) correlation measure for this bicluster is defined as:

5 $$AKR\left( {{\text{M}}',~{\text{N}}'} \right) = 2*\max \left\{ {\frac{{\mathop \sum \nolimits_{{i \in M^{\prime}}} \mathop \sum \nolimits_{{j \ge i + 1,j\smallint M^{\prime}}} \tau _{{ij}} }}{{\left| {M^{\prime}} \right|\left( {\left| {M^{\prime}} \right| - 1} \right)}},\frac{{\mathop \sum \nolimits_{{k \in N^{\prime}}} \mathop \sum \nolimits_{{l \ge k + 1,k\smallint N^{\prime}}} \tau _{{kl}} }}{{\left| {N^{\prime}} \right|\left( {\left| {N^{\prime}} \right| - 1} \right)}}~} \right\}$$

$\:\tau\:$_*i*, *j*_ $\:\tau\:$_*k*_ Here, *i*, *j* (*i* ≠ *j*) is Kendall’s rank correlation for the rows i and j of the bicluster (M′, N′). *k*, *l* (*k* ≠ *l*) represents Kendall’s rank correlation for the columns *k* and *l* of the same bicluster. (M′, N′). The AKR is bounded by:

6 $$\:-1\le\:AKR\left({M}^{{\prime\:}},N{\prime\:}\right)\le\:1$$

Kendall’s rank correlation ranges from − 1 to 1, where values near either end indicate a strong relationship between the two vectors. Similarly, the Average Kendall’s Rank (AKR) also ranges from − 1 to 1. When the AKR value is near either end of this range, it means that the genes or conditions within the bicluster are highly correlated.

**Fig. 2:**
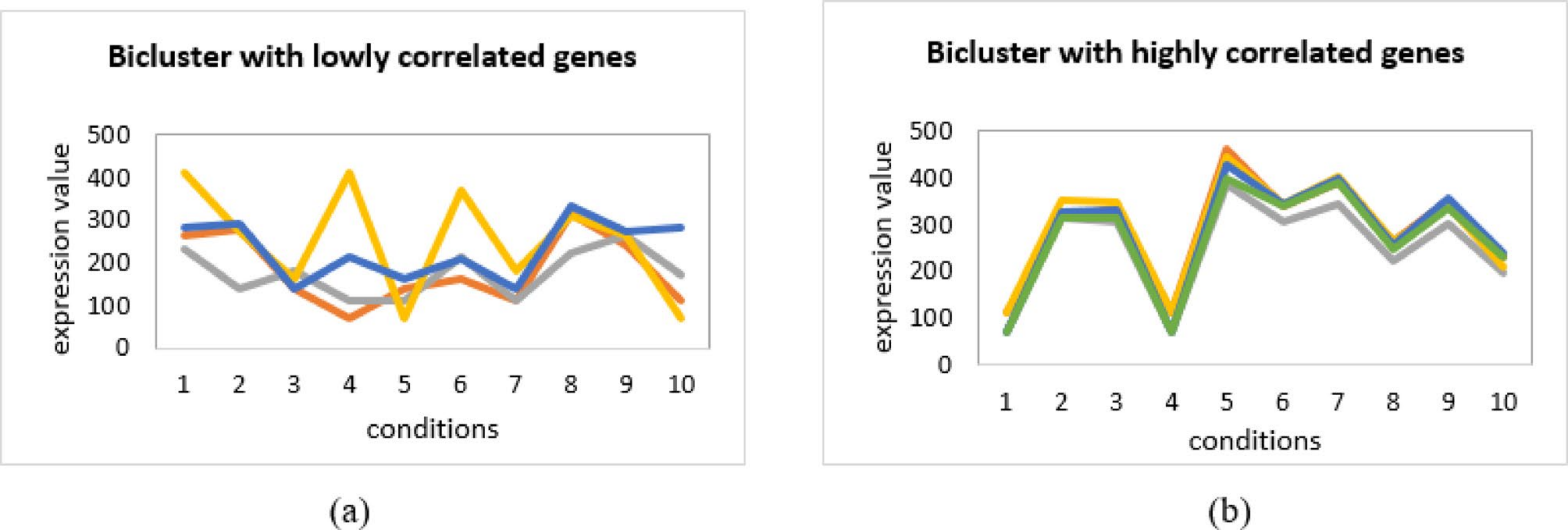
Expression profiles of biclusters: (**a**) genes with weak correlations, (**b**) genes with strong correlations.

The figure compares two biclusters. In Fig. 2a, the genes show low correlation. Their expression values vary differently across the conditions, so the overall correlation is very small (0.001). In Fig. 2b, the genes show high correlation. Their expression values follow a similar pattern across the conditions, giving a high correlation value (0.95).

Biclusters with large volume and highly associated genes are favored in this study. To evaluate both the uniformity and the size of a bicluster B, the Coherence Index (CI) is used as an assessment measure^28^. The CI is defined as the proportion between the AKR value and the size of the produced bicluster. Accordingly, the quality of a bicluster can be measured through the following fitness equation:

7 $$\:ft\left(B\right)=\left(1-AKR\left({M}^{{\prime\:}},N{\prime\:}\right)\right)+CI$$

Biclusters are considered optimal when they yield the lowest fitness function value. Therefore, the term (1 *– AKR(M’*,*N’)*)) is used to select biclusters that contain genes with strong correlations.

### Black-hole representation

In this method, each black hole acts as a solution to the optimization task. Each solution is represented by a binary vector whose length is X + Y, where X corresponds X + Y, where X is the number of rows and Y is the number of columns in the microarray expression data. If a bit is set to 1, the corresponding row or column is included in the bicluster; if it is 0, it is excluded. The mapping function from a black hole to its binary string representation of a bicluster is given in Eq. (8).

8 $$y_{{ij}} \, = \,\left\{ \begin{gathered} x_{{ij}} \, < \,\,0.5\,\,\,\,\,\,\,\,\,\,\,\,\,\,\,\,\,\,0 \hfill \\ otherwise\,\,\,\,\,\,\,\,\,\,\,\,\,\,\,1 \hfill \\ \end{gathered} \right.$$

where.

*x*_*ij*_ - Random value generated between 0 and 1 for *j*^*th*^ gene/condition of *i*^*th*^ point.

*y*_*ij*_ - Binary string representation of bicluster of *x*_*ij*_.

In *y*_*ij*_, if a bit is set to 1 then the corresponding gene or condition belongs to the encoded bicluster; otherwise, it is not.

**Fig. 3:**
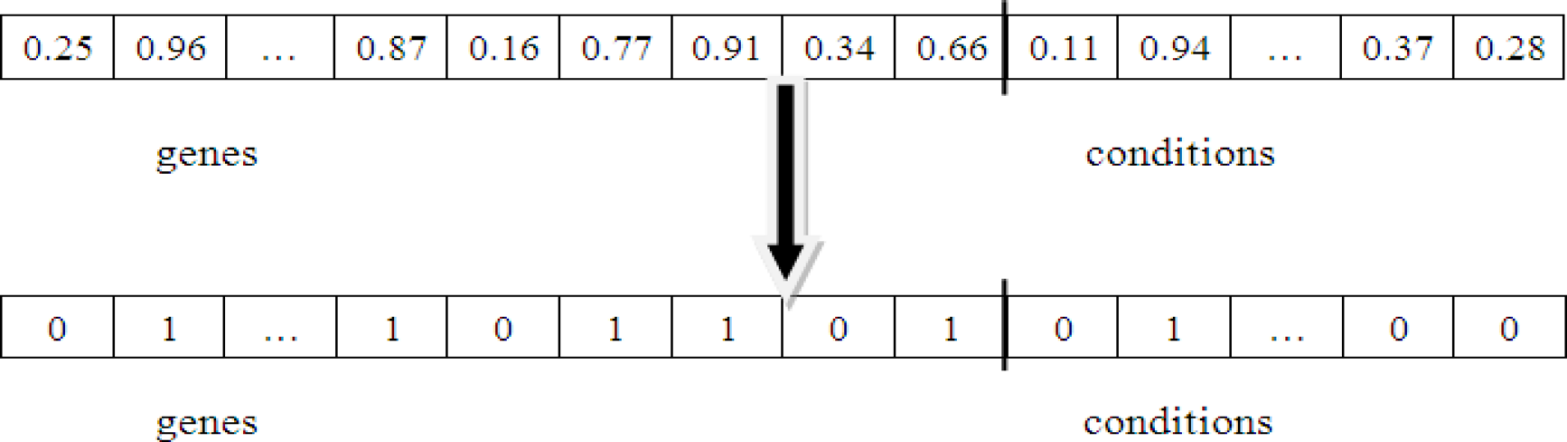
View of encoding from black hole into bicluster.

Figure 3 shows the mapping of a black hole population into a bicluster. The solution is first expressed as a sequence of real numbers, where the initial part refers to genes and the later part refers to conditions. These values are then converted into a binary sequence. This process provides a straightforward way to represent biclusters from candidate solutions.

## Modified stellar mass black-hole optimization

Meta-heuristic algorithms depend on two main properties: diversification and intensification. Diversification, or exploration, helps the algorithm search across various regions in the searching space. Intensification, considerations on improving the best solutions found so far. A proper balance between these two is necessary for good performance. The SBO algorithm has strong global search ability. However, it often suffers from premature convergence and may fail to reach the best solution. To overcome this problem, an improved version called the MSBO biclustering algorithm is proposed.

To overcome this problem, an improved version called the MSBO biclustering algorithm is proposed. It employs Lévy Flight in conjunction with the Nelder–Mead simplex method to achieve a more equitable balance between exploration and exploitation. Nelder–Mead method refines the search near good solutions, while Lévy Flight introduces random jumps to explore new areas. This combination helps that the SBO is able to stay out of local optima. To assess the coherency of extracted solution, the AKR metric is used. AKR measures the coherence of biclusters by detecting scaling and shifting patterns between genes and conditions. Using this approach, MSBO can identify multiple meaningful biclusters from a gene expression data matrix.

To make the proposed method easier to follow, we first describe the goal of bicluster formation before explaining the algorithm. Gene expression data can be denoted as an *X×Y* matrix *P* containing real numbers. Let *R* denote the set of genes and, *C* a set of conditions, and *P(R*,* C)* representing the expression matrix where *R={1*,*2*,*…*,*x}* and *C={1*,*2*,*…*,*y}*. Each cell value *RCx*_*i, j*_ of *P(R*,* C)* symbolises the expression level of gene ‘*i*’ under condition ‘*j*’. The objective of biclustering algorithm is to discover the sub-matrix *B(R’*,* C’)* of *P(R*,* C)*, which is identified by gene subset *R’* $\subseteq$ *R* and condition subset *C’* $\subseteq$ *C*. In general, the problem involves identifying large sets of rows and columns (i.e., high-volume submatrices) that show unexpected similarities along the specified dimensions. The volume or cardinality of a bicluster is given by the product of the number of genes and the number of conditions it contains. In this work, the primary objective is to find the largest biclusters that maximize both the Average Kendall’s tau Rank (AKR) and the row variance.

Evolutionary algorithms are more effective and suited than other deterministic heuristic approaches because they may investigate vast and intricate sets of possible solutions to identify optimal or nearly optimal outcomes. The SBO approach has now been successful in solving the majority of engineering’s complicated optimization issues^25^. In the black hole optimization method, a solution’s success mainly depends on its mass. The mass is influenced by how much it absorbs and emits. The algorithm copies the idea of natural selection. It keeps the best solutions and makes new ones from them. This process guides the group toward better results. SBO uses this approach to find the best values of the objective functions. However, the basic form is often inefficient and may converge slowly on difficult problems.

Nelder and Mead designed the simplex method to search for a local minimum among a group of values^26^. The term simplex is used since a triangle can be generalized to any number of dimensions. In two dimensions, this shape is a triangle. The method works by checking and comparing the function values at the three points of the triangle, named a, b, and c. The worst vertex is the one at which f (a, b) has the maximum value It is excluded from the set and replaced with a new one. The search goes on as a new triangle with various shapes is generated. The function values at the vertex points decrease over time. The triangles are shrunk in size, and the minimum point’s coordinates are discovered. It works well and is computationally efficient. An initial population of three people is chosen in this modified SBO; this population is slightly bigger than that in normal SBO. The optimal initial population is then achieved by doing all the simplex Nelder-Mead method’s required operations. The most suited individual is selected in each iteration. Then, a new population is produced using the best initial answer. To find a new solution, the optimization process is then repeated using regular SBO with a smaller population.

The so-called adoption of the simplex approach is a fascinating local search capability proposed by the SBO algorithm. Despite having several intriguing features, SBO has certain drawbacks in terms of evolutionary computing. Although this method is appropriate for local exploitation, excessive evaluation of candidate solutions can significantly raise the computational cost of the algorithm, a drawback common to nearly all optimization techniques. For instance, SBO improves its initial random solutions by performing random walks around several of the best candidates during selection. Despite the fact that this process may be advantageous in some circumstances, it results in an imbalance between intensification and diversity, which may have a substantial impact on the core meta-heuristic principle. Levy flight is thus included in order to get around the aforementioned problem and increase the standard SBO’s optimization effectiveness. Levy flight is used by the majority of meta-heuristic algorithms to solve global optimization by intensifying and diversifying their search space. A fair balance is created between exploration and exploitation as a result^29^. In SBO, exploration is accomplished by combining a deterministic process with randomization. The freshly created solutions are then dispersed throughout the issue search area in a variety of ways. A uniform or Gaussian distribution is used in typical SBO to achieve randomization. But there are other ways to achieve randomization. Lévy distributions^30^ have been more popular in recent years as an alternative to uniform or Gaussian distributions. Equation (9) defines the Lévy flight employed to generate the new solution *x(t + 1)* for black hole *i*.

9 $$x_{i} (t + 1) = \,x_{i} (t)\, + \,\alpha \,Levy(\lambda )$$

A multiplication of entries is represented by the symbol ⊕. Equation (10) provides a Levy distribution for large steps, from which Levy flights derive their random steps. In essence, Levy flights offer a random walk.

10 $$Levy\sim u = t^{{ - \lambda }}$$

The following are the key components of the suggested work: It is a population-based algorithm where the optimal black hole is chosen using the simplex approach from the original random population and adjustments to the structure of new individuals are made utilizing levy distribution operators.

**Fig. 4:**
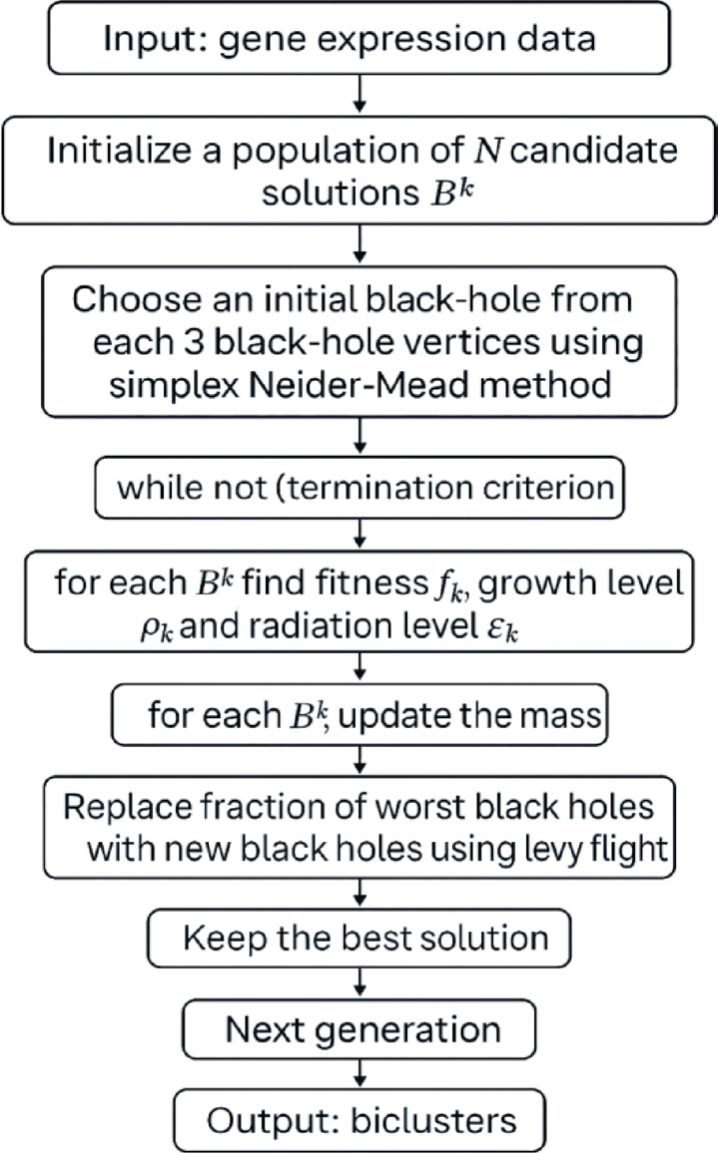
Flowchart of modified SBO.

This Fig. 4 illustrates process of applying the Modified SBO algorithm to microarray data for biclustering. The algorithm starts by initializing a population of candidate solutions representing black holes. An initial black hole is selected from every three vertices using the Nelder-Mead method. During each iteration, the fitness, growth level, and radiation level of each black hole are computed, and their masses are updated accordingly. A portion of the weakest black holes is replaced through Lévy flight to maintain exploration. The best solution is retained in every generation, and the process runs until the stopping condition is reached. In the end, the method produces a set of highly correlated genes and samples from the input matrix.

## Experiemental results

### Performance analysis of benchmarks functions

There isn’t a specific guideline for determining the best optimization function to use when assessing the performance of the proposed approach. Most frequently, the optimum value is used to assess the MSBO method’s performance. The 10 benchmark functions listed in Table 1 were utilized to evaluate the effectiveness of MSBO with SBO and Cuckoo Search Optimization (CSO)^31^. These benchmark factions, which range in complexity, are frequently used in comparative investigations. This kind of benchmark function can measure how well an algorithm balances intensification and diversification. The benchmark functions are thought to have 30 dimensions. Each of the benchmark functions’ global minimum values is calculated using a different initial population after 10 iterations. The population is 50. The optimal check is reached or the maximum iteration of 1000 is the stopping requirement. We employed ten benchmark functions that were unimodal, multimodal, separable or non-separable, convex, and continuous to assess the performance and sustainability of the presented selection operators. It’s interesting to note that the empirical experiments adopted the default parameter settings for SBO and CSO^25,32^. The parameter settings for the algorithms are given below, and all experiments were implemented in MATLAB. These settings play an important role, as they determine how quickly the algorithm converges, how well it balances exploration and exploitation, and how effectively it performs overall. Selecting suitable values was therefore an essential part of the experimental setup.

**Table 1:** Benchmark functions used in experiments D: Dimension, C: Characteristics, U: Unimodal, M: Multimodal, S: Separable, N: Non-separable.

S.no.	Range	D	C	Function
1	[-600,600]	30	MN	Griewank
2	[-32,32]	30	MN	Ackley
3	[-10,10]	30	UN	Dixonprice
4	[-5.12,5.12]	30	MS	Rastrigin
5	[-30,30]	30	UN	Rosenbrock
6	[-500,500]	30	MS	Schwefel
7	[-10,10]	30	US	Sum squares
8	[-100,100]	30	US	Sphere
9	[-100,100]	30	US	Step
10	[-1.28,1.28]	30	US	Quartic

Table 2 shows the performance of MSBO on ten benchmark functions (F1–F10). For each function, we report the global optimum, optimum value, mean and standard deviation across runs, and average number of iterations. In the case of F7, the theoretical optimum is − 50, and the value found by MSBO (–50.000000000000128) matches it to a very high degree of precision. The small standard deviations suggest stable behavior across runs. The iteration count required varies according to the function’s level of complexity. Simple cases like F1, F4, and F9 reach convergence within a few dozen iterations, while harder functions such as F6 and F7 require several hundred or more. Overall, the results indicate that MSBO provides accurate and consistent solutions across different types of problems.

**Table 2:** Optimization results of MSBO on standard benchmark functions (Best, Mean, Std, Iterations).

S.no	Function name	Optima check	Best optima	Std (mean) optima	Mean optima	Mean of iterations
1.	F1	0	0.0000000000000000	0.0000000000000000	0.0000000000000000	32.25
2.	F2	0	0.0000000000000006	0.0000000000000009	0.0000000000000026	27.6
3.	F3	0	0.0000000000000011	0.0000000000000002	0.0000000000000039	59
4.	F4	0	0.0000000000000000	0.0000000000000000	0.0000000000000000	28.95
5.	F5	0	0.0000000056734570	0.0000000314568829	0.0000000984899237	108.19
6.	F6	0	0.0000000000000019	0.0000000000000003	0.0000000000000005	672.65
7.	F7	-50	-50.000000000000128	-50.000000000000772	-50.000000000001007	3728.7
8.	F8	0	0.0000000000000000	0.0000000000000000	0.0000000000000000	439.2
9.	F9	0	0.0000000000000000	0.0000000000000000	0.0000000000000000	300.00
10.	F10	0	0.0000000000000000	0.0000000000000000	0.0000000000000000	44.64

**Fig. 5:**
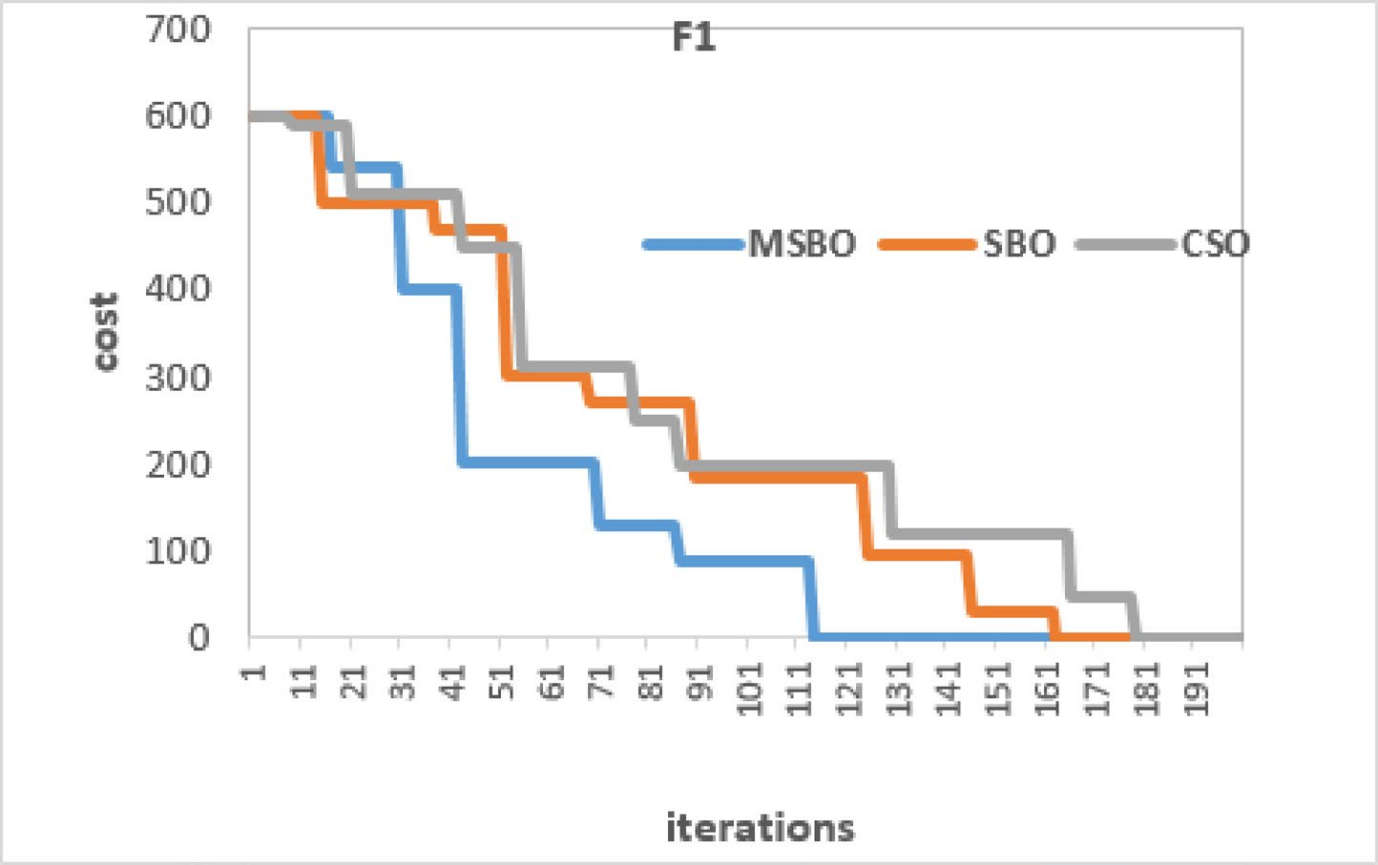
Convergence curves of MSBO, SBO, and CSO on test function F1.

**Fig. 6:**
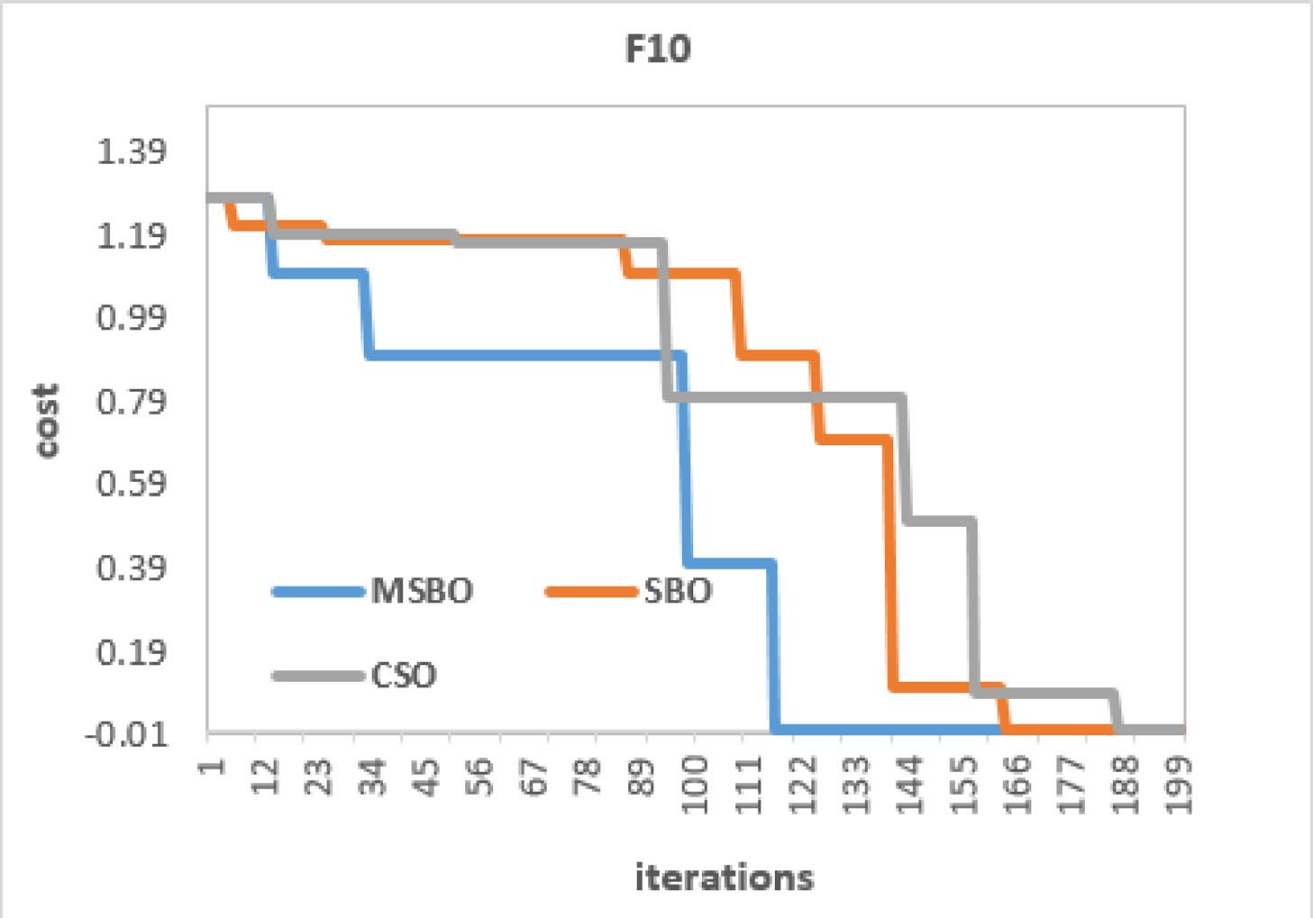
Convergence Curves of MSBO, SBO, and CSO on Test Function F10.

Figures 5 and 6 present the convergence performance of MSBO, SBO, and CSO on two representative benchmark functions. F1 (unimodal) and F10 (multimodal) are selected to illustrate the behavior of the algorithms on different types of test functions. In both cases, the cost represents the global best fitness value, and the stopping criterion is set at a maximum of 200 iterations. For the unimodal function F1 (Fig. 5), the MSBO reached the global optimum (0.0) within approximately 120 iterations, whereas SBO and CSO required closer to 150–170 iterations. The faster convergence of MSBO indicates its superior exploitation ability on unimodal landscapes. The convergence curve of MSBO shows a steeper decline in the early stages, demonstrating its ability to reduce the fitness value more quickly compared to SBO and CSO.

For the multimodal function F10 (Fig. 6), MSBO performed better than SBO and CSO. It reached a cost close to zero in about 140 iterations, while the other two methods needed 160–180 iterations for similar results. In both unimodal (F1) and multimodal (F10) cases, MSBO converged faster and with higher accuracy. On average, it reduced the number of iterations by around 20–25% for F1 and 15–20% for F10, while keeping results very close to the global optimum.

The Table 3 compares the performance of three algorithms MSBO, SBO, and CSO on benchmark functions F1 through F10 using three evaluation measures: best, mean, and standard deviation of the mean optimum (Std). It highlights the best results (in bold) obtained for each benchmark function. The findings demonstrate that CSO beats SBO in performance on the last four unimodal separable functions (F7–F10). However, MSBO consistently achieves stronger performance overall. This indicates that while SBO shows good convergence speed and exploitation ability, it falls behind CSO in terms of exploration. By contrast, MSBO achieves better results than both SBO and CSO on several unimodal and multimodal functions (such as F2, F3, F6, F7, and F10). For the remaining functions, although its performance is not always the very best, MSBO still maintains competitive outcomes. The outcomes indicate that the hybrid approach is able to preserve global optimum solutions, thereby minimizing the risk of premature convergence. With the exception of F5, MSBO consistently achieves stable and reliable performance across the benchmark functions, highlighting its robustness. Taken together, these results establish MSBO as the most effective method among the three algorithms evaluated.

**Table 3:** Performance Comparison of MSBO, SBO, and CSO on Benchmark Functions (F1–F10).

No	Best optimum			Mean optimum			Std(mean) optimum		
	MSBO	SBO	CSO	MSBO	SBO	CSO	MSBO	SBO	CSO
F1	0.0000000000000000	0.0000000000000000	0.0000000000000000	0.0000000000000000	0.0000000000000000	0.0000000000000000	0.0000000000000000	0.0000000000000000	0.0000000000000000
F2	**0.0000000000000006**	0.0000000000000159	0.0000000000034166	0.0000000000000026	0.00000000000002484	0.0000000000049877	0.0000000000000009	0.0000000000000887	0.0000000000034166
F3	**0.0000000000000011**	0.0000000000000068	0.0000000005667768	0.0000000000000039	0.0000000000001255	0.0000000028712598	0.0000000000000002	0.0000000000000368	0.0000000013677769
F4	0.0000000000000000	0.0000000000000000	0.0000000000000000	0.0000000000000000	0.0000000000000000	0.0000000000000000	0.0000000000000000	0.0000000000000000	0.0000000000000000
F5	0.0000000056734570	**0.0000000000034570**	0.0000045677134570	0.0000000984899237	0.0000000000064356	0.0000076698124000	0.0000000314568829	0.0000000000003090	0.0000095566781129
F6	**0.0000000000000019**	0.0000000000000034	0.0000000000003452	0.0000000000000005	0.000000000001256	0.0000000000782349	0.0000000000000003	0.0000000000000647	0.0000000000013543
F7	**-50.000000000000128**	-50.0000000000065892	-50.0000000000004518	-50.000000000001007	-50.00000000007654420	-50.0000000000096190	-50.000000000000772	-50.000000004199548	-50.000000000166398
F8	0.0000000000000000	0.0000000000000000	0.0000000000000000	0.0000000000000000	0.0000000000000000	0.0000000000000000	0.0000000000000000	0.0000000000000000	0.0000000000000000
F9	0.0000000000000000	0.0000000000000000	0.0000000000000000	0.0000000000000000	0.0000000000000000	0.0000000000000000	0.0000000000000000	0.0000000000000000	0.0000000000000000
F10	**0.0000000000000000**	0.0000000000000822	0.0000000000000045	0.0000000000000000	0.0000000000002070	0.0000000000000515	0.0000000000000000	0.0000000000000128	0.0000000000000059

### Performance evaluation of microarray data

This section presents an evaluation of the proposed MSBO algorithm using synthetic datasets and actual gene expression data. Its performance is benchmarked against four widely recognized biclustering methods: CC^8^, ISA^9^, OPSM^10^, and Bimax^11^. All comparisons were conducted using the BicAT^33^. It is comprehensive platform that integrates multiple biclustering algorithms and facilitates clustering-based data analysis.

#### Analysis of synthetic data

Following the method described in^12,13^, a synthetic dataset of size (200, 20) was generated.The datasets included various bicluster types, including constant pattern, shifting, scaling, and coherent behavior. To ensure statistically reliable results, ten problem instances were created for each dataset type in arbitrarily placing patterns not at same positions within input data. Consistent with^12^, employed two evaluation ratios to determine the performance of the proposed biclustering approach:

11 $$\:{\theta\:}_{Shared}=\frac{{S}_{cb}}{{Tot}_{size}}\times\:100$$

12 $$\:{\theta\:}_{Notshared}=\frac{{S}_{ncb}}{{Tot}_{size}}\times\:100$$

The variables are defined as follows: S_cb_ measures the correctly extracted portion of biclusters, S_ncb_ quantifies the incorrectly extracted portion and Tot_size_ to the overall size of the true biclusters.

The ratio *θ*_*Shared*_ ​ (respectively, *θ*_*NotShared*_) represents fraction of bicluster volume that corresponds (respectively, does not correspond) to the true implanted biclusters. A value of *θ*_*Shared*_ = 100% indicates that the algorithm has perfectly extracted the true biclusters, while, *θ*_*NotShared*_ = 100% means that all extracted biclusters are incorrect. Thus, the ideal outcome is *θ*_*Shared*_ = 100% and *θ*_*NotShared*_ = 0%. For each algorithm, we compute both measures to report the average percentage of bicluster volume that overlaps with, or deviates from, the true implanted biclusters. The goal of this evaluation is to identify which algorithm can most effectively recover all implanted biclusters.

**Table 4:** Performance analysis of MSBO with other State of art methods for synthetic data

Algorithms	θ _Shared_ (%)	θ _NotShared_ (%)
CC	18.21	36.57
OPSM	46.39	74.42
ISA	39.38	5.31
Bimax	58.18	21.39
MSBO	70.24	34.17

As shown in Table 4, the MSBO demonstrates superior performance by successfully extracting 70.24% of the implanted biclusters, with an additional volume representing 34.17% of the implanted biclusters. This occurs because, in some cases, combining two biclusters introduces extra volume only under specific conditions, but still preserves the exact number of genes. In comparison, the best-performing existing algorithm, Bimax, extracts only 58.18% of the implanted biclusters and produces an additional volume of 21.39%.

The differences in performance among competing algorithms are rooted in their design strategies. CC adopts the MSR function as its biclustering criterion. However, when the signal of implanted biclusters is weak, its greedy nature tends to eliminate rows and columns early in the process, preventing their recovery in the final biclusters. ISA focuses exclusively on constant up-regulated and down-regulated expression values. As a result, it may fail to capture coherent biclusters, often missing some rows and columns. OPSM is limited to detecting up- and down-regulation patterns with coherent evolution, which leads to reduced effectiveness in scenarios involving constant biclusters. Finally, Bimax, which relies on discretization preprocessing, cannot adequately capture the elements within coherent biclusters and therefore struggles to accurately identify implanted biclusters.

#### Analysis of benchmark data

 The performance of the suggested MSBO approach was assessed using two benchmark datasets. 2,884 genes’ expression under 17 conditions is recorded in the first dataset, which is derived from the yeast cell cycle. The second one comes from human B-cell lymphomas, containing the expression of 4,026 rows across 96 different samples. We applied two main criteria to assess the performance of the proposed biclustering technique. The analysis began by estimating p-values to determine the robustness and significance of the discovered biclusters. This was followed by evaluating their biological annotations to gauge functional relevance.

The Table 5 presents details of five representative biclusters chosen from the yeast and lymphoma datasets. The table lists a few biclusters that were randomly chosen from 100 possible ones. Here, the first column specifies the bicluster ID. Columns two and three display the number of genes and conditions. Column four gives the bicluster volume, and column five lists the Average Kendall’s Rank (AKR) value. The sixth column presents the Coherence Index (CI). The size shows the overall volume of the bicluster, while AKR measures how consistent the gene–condition relationships are, and CI reflects the coherence quality. In the yeast cell cycle data, the largest bicluster was found with an AKR of 0.89. Although its coherence index (CI) was relatively low, it still reflects the strength of the partitioning. The lowest CI value observed was 0.0026 for a bicluster of size 340. In the lymphoma dataset, the largest bicluster had 779 elements, with an AKR of 0.81 and a CI of 0.0010. As mentioned earlier, a higher AKR value shows that the detected biclusters are strongly coherent.

**Table 5:** Characteristics of five representative biclusters selected from 100 biclusters in the yeast Cell-Cycle and lymphoma datasets, including gene and sample counts, bicluster size, average Kendall rank (AKR), and confidence index (CI).

ID	genes	Samples	size	AKR	CI
YBC2	26	9	234	0.94	0.0040
YBC15	34	10	340	0.89	0.0026
YBC18	38	8	304	0.92	0.0030
YBC54	27	9	243	0.93	0.0038
YBC77	32	10	320	0.89	0.0027
Lymphoma dataset					
LBC12	34	18	612	0.89	0.0014
LBC28	32	21	672	0.86	0.0012
LBC35	35	20	700	0.84	0.0012
LBC42	41	19	779	0.81	0.0010
LBC69	33	20	660	0.87	0.0013

**Fig. 7:**
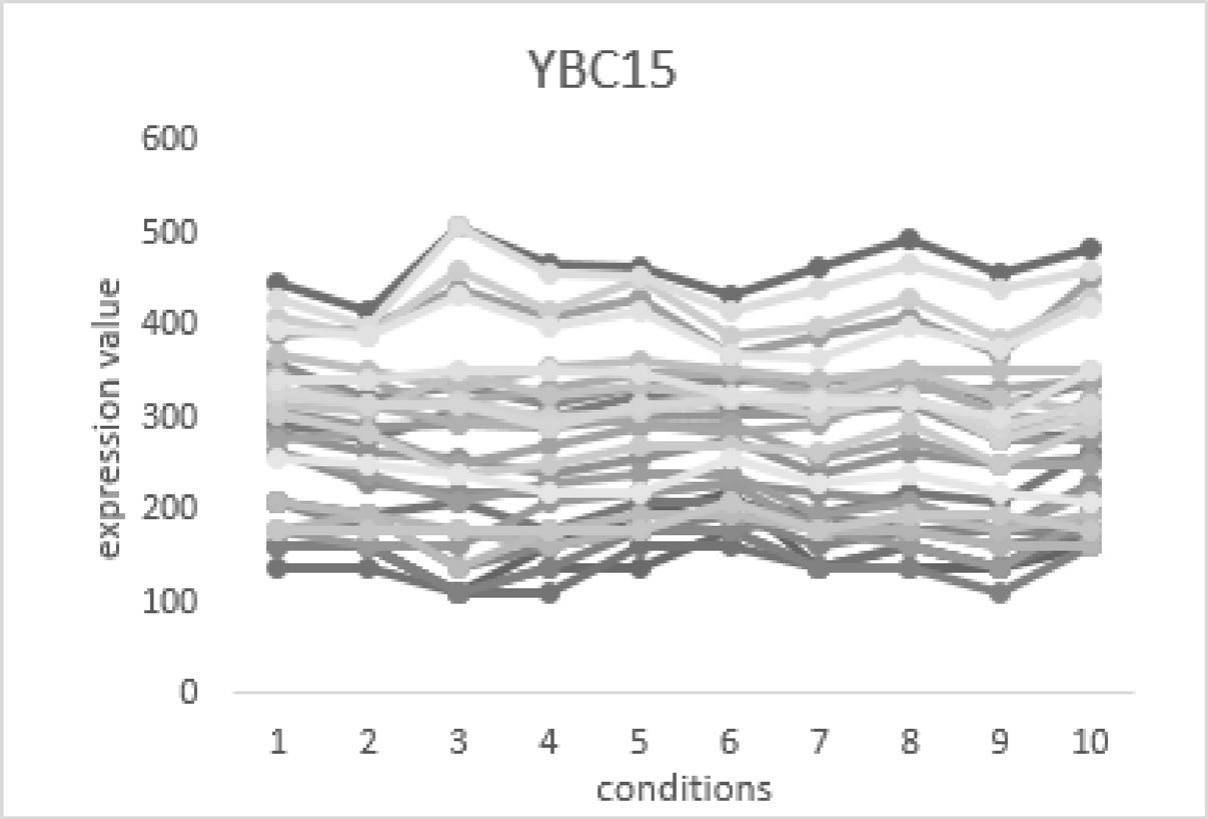
Expression profile of bicluster YBC15 (34 genes × 10 conditions) from yeast cell-cycle data.

Figure 7 shows the bicluster YBC15, which consists of 34 genes evaluated across 10 experimental conditions, giving a bicluster size of 340. The expression profiles of these genes are plotted against the conditions. Most of the genes show clear up- and down-regulation patterns. This indicates that they are co-regulated under the given conditions. The AKR value of 0.89 reflects strong coherence among the genes. It means their expression patterns are highly consistent. The Coherence Index (CI) of 0.0026 is very low. This suggests that noise or irregular variations within the bicluster are minimal. Together, these results show that YBC15 is a biologically important bicluster. It captures a group of genes with stable and coordinated expression during the yeast cell cycle.

###  Assessing Biological Importance Using GO enrichment analysis

Scientists have recently put a lot of effort into this specific biological study because they’ve recognized how important biclustering applications are. This study gives a detailed look at how biclustering is mainly used in biological data. A p-value is essential for finding significantly overrepresented functions. It represents the probability that the genes in a cluster were chosen by chance. The unique features of the cluster were not coincidental, as indicated by a low p-value.One important bioinformatics application is calculating the likelihood that a specific number of genes from a Gene Ontology category (e.g. component, process, or function) show up in every bicluster^34^. One of the most common ways to evaluate a biclustering method is by its GO-based significance. This approach, which relies on statistically significant GO annotations, demonstrates how strongly a gene group obtained through biclustering is enriched in a specific GO category.

**Fig. 8:**
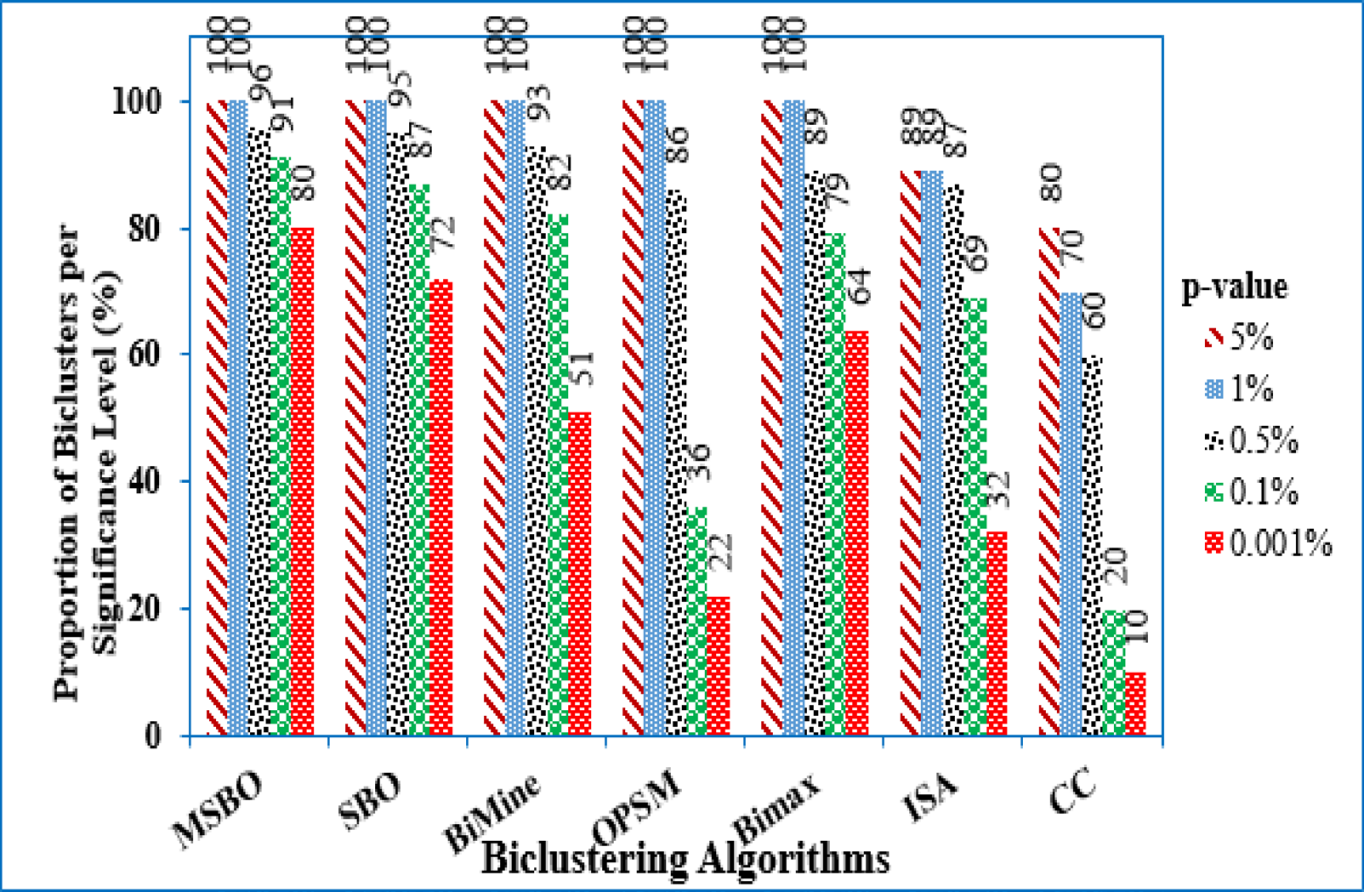
Enriched GO annotations for biclusters in yeast microarray data

The biclusters discovered in the Gasch Yeast dataset are biologically meaningful when evaluated using the proposed nature-inspired method. The proposed method’s biological relevance for the yeast cell-cycle dataset was examined by benchmarking its results against Bimax, ISA, CC, OPSM, and BiMine^13^. This analysis employed the FuncAssociate 2.0 web-based application^35^, which assigns adjusted significance scores to every bicluster. These scores correspond to p-values indicating how strongly the genes within a bicluster are associated with various GO categories. Smaller p-values, closer to zero, suggest a stronger and more reliable match. The distribution of p-values for each algorithm across all extracted biclusters is shown in Fig. 8. In our results, all biclusters identified by MSBO had p-values ranging from 5% to 1%. Notably, 80% of the biclusters extracted by MSBO had p-values of 0.001% or lower, compared to 72% for SBO, 69% for Bimax, and at most 51% for CC, ISA, and OPSM. These results demonstrate that MSBO consistently outperforms CC, ISA, Bimax, and OPSM, not only for the stringent p-value of 0.001% but across all evaluated significance levels (5%, 1%, 0.5%, 0.1%, and 0.001%).

#### Functional activity analysis

One of the three structured vocabularies is the molecular function vocabulary. It stands for fundamental processes like catalysis or binding. We made use of the GOTermFinder tool from the Saccharomyces Genome Database (SGD). This work employed the GOTermFinder tool available through the Saccharomyces Genome Database. It further displays to users the possible shared characteristics among the genes. With GOTermFinder, you can find important GO terms shared by sets of genes. Table 6 provides a summary of the significantly overrepresented GO terms for every bicluster, classified under cellular component, molecular function, and biological process. To enhance readability, we report only the most significant terms. As an illustration, a large proportion of the genes in bicluster YBC15 are linked to structural molecule functions. As denoted by (n = 17, p = 8.19 × 10^− 9^), 17 of the 34 genes in this bicluster exhibit a strong association with this function, supported by high statistical significance. Such enrichment patterns highlight the biological coherence of the identified biclusters.

**Table 6:** Major GO annotations for three biclusters derived from yeast cell analysis.

ID	No. of genes	Process	Function	Component
YBC15	34	Cellular Process ( *n* = 18, *p* = 5.66 × 10^− 13^)	Structural Molecule Activity (*n* = 17, *p* = 8.19 × 10^− 9^)	Cell Part (*n* = 17, *p* = 2.35 × 10^− 13^)
YBC77	32	Cellular Component Organization ( *n* = 17, *p* = 3.73 × 10^− 16^)	Binding Activity (*n* = 14, *p* = 5.92 × 10^− 12^)	Nuclear Part (*n* = 19, *p* = 4.63 × 10^− 14^)
YBC18	38	Metabolic Process ( *n* = 21, *p* = 2.28 × 10^− 12^)	Hydrolase Activity ( *n* = 16, *p* = 7.91 × 10^− 8^)	Intracellular Part (*n* = 20, *p* = 5.47 × 10^− 15^)

**Fig. 9:**
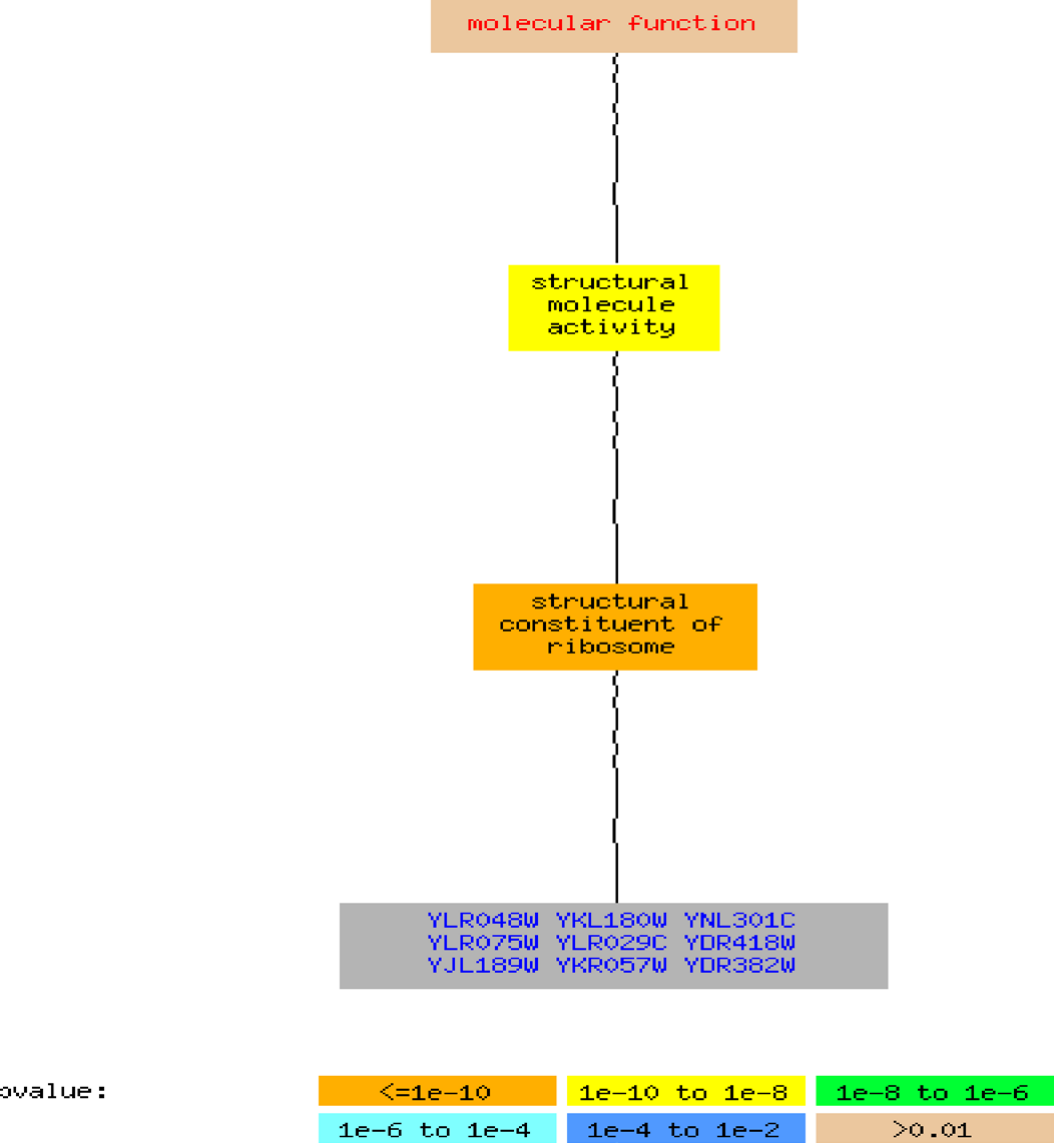
GO molecular function for yeast cell cycle MSBO results (9 genes).

The biological network of the yeast cell-cycle data containing nine genes is presented in (Fig. 9). The nodes in the diagram are shaded according to their p-values. Each gene is linked to the GO term with which it is directly associated. To keep the graph brief, only significantly enriched terms with a p-value of 0.01 or lower, along with their descendant terms, are displayed. The marked genes (YLR048W, YKL180W, YNL301C, YLR075W, YLR029C, YDR418W, YJL189W, YKR057W, and YDR382W) are directly related with ribosomal structure and protein synthesis. The enrichment pattern recommends that this proposed bicluster approach captures a biologically coherent group of genes that contribute to the fundamental process of translation, which is central to cell-cycle regulation in yeast. For example, the GO term “structural constituent of ribosome” was significantly enriched, with 4 of 9 genes (44.4%) in the bicluster associated with this function, compared with 227 of 7,166 genes (3.2%) in the genome background. This enrichment corresponds to a corrected p-value of 0.00141, with 0% False Discovery Rate (FDR) and no false positives. The annotated genes include YDR418W, RPL18B, RPP2B, and RPL39, which encode ribosomal proteins essential for protein synthesis and cellular growth. Their enrichment suggests that MSBO successfully captures biclusters representing fundamental biological processes, particularly ribosome-related functions. Additionally, the associated p-value is extremely small, indicating that there is very little chance that the gene cluster will emerge at random. These findings indicate that the proposed MSBO biclustering approach is capable of discovering biologically significant biclusters.

## Conclusions

We present in this research a Modified Stellar Mass Black-hole Optimization (MSBO) technique, which uses Average Kendall correlation measure to locate biclusters in expression data. In addition, to find biclusters the suggested approach uses a Levy flight and the Nelder-Mead simplex strategy. The proposed method uses the Lévy flight mechanism to regulate and stabilize population size. In addition, the simplex technique helps expand the population, accelerate convergence, and generate new solutions with enhanced exploration and exploitation of the search space. The method was tested on yeast and human Bcell expression data, and its results were compared to those from other approaches. The analysis showed that the genes grouped in our biclusters were more strongly enriched with Gene Ontology (GO) terms and produced better p-values than those generated by traditional algorithms. Among the approaches studied, MSBO proved to be one of the most effective, as it identified a larger proportion of enriched biclusters in real datasets. However, when applied to very large RNA-seq datasets containing tens of thousands of genes, the method can become computationally demanding. Future work will focus on improving the algorithm so it can manage massive amounts of data. A potential direction is to use parallel processing for correlation and fitness calculations, which would enhance scalability and maintain the method’s usefulness for modern high-throughput transcriptomic investigation.

## Data Availability

All datasets used in this study were collected from public repository.
